# Editorial: The costs of caring for older adults

**DOI:** 10.3389/fpubh.2026.1942774

**Published:** 2026-08-11

**Authors:** Lawrence Ejike Ugwu, Matthew Aplin-Houtz, Janine Anthea White

**Affiliations:** 1 Faculty of Humanities Mafikeng, North-West University, Mmabatho, South Africa; 2 Centre for Applied Psychology and Public Health Research in Africa, Enugu, Nigeria; 3 Department of Psychology, Brooklyn College, New York, NY, United States; 4 Faculty of Health Sciences, University of the Witwatersrand Johannesburg, Johannesburg, South Africa

**Keywords:** care givers, community support, digital technologies, financial implications, integrated care, older adults, social determinants

Population aging is reshaping health systems, households, labor markets and communities across the world. Longer life expectancy represents a major public health achievement, but it also raises urgent questions about how care is organized, financed, delivered and sustained. The articles in this Research Topic show that the costs of caring for older adults cannot be understood through healthcare expenditure alone. They include the visible costs borne by health systems and households, but also the less visible costs of unpaid caregiving, fragmented services, digital exclusion, social inequality, functional decline and limited access to appropriate support. Collectively, the contributions invite a broader understanding of care as a shared social, economic, relational and policy responsibility.

Across the 36 papers in this Research Topic, cost emerges not as a single financial measure, but as a layered concept shaped by health, family, social, economic and policy conditions. The studies examine diverse populations, care arrangements and national contexts, yet they converge on a common message: caring for older adults requires systems that recognize the interdependence of clinical need, household resources, caregiver capacity, community support and policy design. The Research Topic therefore moves the discussion beyond the question of how much aging costs to ask who carries these costs, why some groups carry more than others, and what forms of care can distribute responsibility more fairly.

The most visible costs of aging are often measured through healthcare expenditure. Several papers quantify the financial implications of chronic disease, disability and acute complications in later life. Evidence on vision loss, delirium and ischemic stroke demonstrates how age-related illness generates direct health-system costs while also threatening quality of life, functional independence and longer-term care needs (Agius et al.; Garcia Morales et al.; Jiao et al.). At the household level, Ugwu et al. extend this discussion by showing how out-of-pocket spending in rural South Africa is shaped by health status, functional limitations and socioeconomic vulnerability. Other contributions broaden the economic lens further: Yang et al. examine the wider market consequences of long-term care insurance, while Li draws attention to the economic exploitation of older adults. Together, these studies show that the financial cost of care is not confined to hospitals or insurers; it is distributed across households, communities, housing markets and social protection systems.

Many of the most consequential costs of caring for older adults remain less visible because they are absorbed by families. Across the Research Topic, informal caregivers appear not as peripheral helpers but as central actors in the care economy. Studies on dementia care, caregiver resilience, health literacy, family functioning and behavioral symptoms illustrate how caregiving demands affect psychological wellbeing, family relationships and time use (Alasqah et al.; Di Nolfi et al.; Liu, Li et al.; Liu, Luo et al.). These pressures are intensified by frailty, disability, demographic change and constrained family structures. Peng et al. synthesize the lived experiences and care needs of frail older patients; Xiang et al. show how China's one-child generation faces structurally imbalanced care responsibilities; and Zhao and Zhao document heterogeneous care preferences among disabled older adults in an urban setting. These articles make clear that supporting caregivers is not only a matter of compassion, but a condition for sustainable health and social care systems.

A further contribution of the Research Topic lies in its attention to the organization of care. Rather than positioning treatment as the sole center of later-life care, several articles call for models that integrate assessment, navigation, community support and social care. Fulceri et al. map comprehensive geriatric assessment and management in primary care, highlighting the need for multidisciplinary teams and coordinated instruments. Coyne et al. illustrate, through a senior care navigator programme, how older adults can be supported to address both health and social needs. Chen, Zhang et al. examine rural demand for socialized older-adult care services, showing how care needs evolve across age, period and cohort influences. At the system level, Guo et al. and Liu, Zhao et al. show that aligning care-service supply with population aging is a complex coordination problem, while Xin and Han caution that community-based integrated care reforms may have unintended consequences, including increased hospitalization expenses. These findings underscore that reform is not automatically beneficial; its effects depend on design, implementation, financing and equity.

Technology offers opportunities to extend care, but the Research Topic appropriately avoids treating digital innovation as a simple solution. Studies of community-based home care and smart home care, digital transformation among “new older adults,” and digital health needs for long-term continuity care demonstrate the promise of technology-enabled support while also exposing persistent risks of exclusion (Chen, Li et al.; Shao et al.; Sun et al.). Digital tools may improve monitoring, communication, self-management and continuity, but their benefits depend on affordability, access, usability and digital literacy. This theme is complemented by Tang et al., whose work on ego depletion and hypertension self-management reminds us that effective care also depends on behavioral resources, motivation and the everyday capacity of older adults to manage chronic illness. Technology, therefore, should be understood as one component of a wider care ecosystem rather than a substitute for human support, social protection or accessible services.

The articles also demonstrate that aging and care are shaped by social determinants and relational environments. Education, socioeconomic position, community context, living arrangements, intergenerational support and social participation influence both health outcomes and care needs. Alberio et al. highlight the role of education and socioeconomic context in health among aging populations. A group of studies on intergenerational relationships and programmes shows that social connection is both a resource for wellbeing and a site where expectations, reciprocity and community environments matter (Cheng, Kwan, et al.; Zhao and Zhu; Cheng, Loo et al.). Zhang et al. add to this discussion by examining mutual-aid care needs among older adults with multimorbidity. Taken together, these contributions caution against viewing older adults only as recipients of care. They also highlight older adults' agency as people who make preferences, sustain relationships, participate in communities and navigate constrained choices.

Place and care environments are equally important. Gil-Lacruz et al. foreground older adults' own perspectives on residential care homes, while Yermukhanova et al. examine the characteristics of older people's needs in Kazakhstan. Zhang and Loukaitou-Sideris compare age-friendly city and community policies, illustrating how built, social and policy environments can either support or restrict healthy aging. Bai et al. focus attention on sleep disorders among older adults in care institutions, reminding us that institutional environments influence everyday wellbeing as well as clinical outcomes. These studies collectively show that care is not delivered in a vacuum. It is embedded in homes, institutions, neighborhoods, cities and policy systems that can either protect older adults' dignity and independence or deepen vulnerability.

The geographical breadth of the Research Topic is a major strength, but its significance lies not only in diversity of setting. Evidence from Georgia, Germany, Poland and China shows how different systems confront shared pressures around workforce capacity, long-term care, mortality risk, chronic illness and access to formal services. Macharadze identifies gaps in geriatric services, education and policy in Georgia; Haß et al. analyse excess mortality among German adults aged 60 years and older who needed long-term care during 2020–2024; Majchrowicz et al. examine the functioning of chronically ill patients receiving long-term care in Poland; and Wu et al. show how urban empty-nest older adults may substitute self-medication for formal medical treatment under dual constraints. These studies demonstrate that the costs of care often appear where formal systems are unavailable, insufficient, poorly coordinated or unaffordable. They also create opportunities for cross-country learning without assuming that one model can be transferred wholesale from one context to another.

Several implications follow from the Research Topic. First, policies should measure and value unpaid caregiving time rather than treating family care as an inexhaustible resource. Second, health and social care should be integrated through comprehensive geriatric assessment, care navigation, community-based services and continuity mechanisms that respond to functional, social and economic needs. Third, reforms should be evaluated not only for intended outcomes but also for unintended consequences, including increased hospitalization, inequitable access or the shifting of costs onto households. Fourth, digital care must be designed around inclusion, affordability, literacy and trust. Finally, aging policy should treat social determinants, household vulnerability, community environments and older adults' preferences as central design concerns rather than background variables.

This Research Topic therefore advances a wider conceptualization of the costs of caring for older adults. The contributions show that these costs extend beyond expenditure to include caregiver strain, fragmented services, digital divides, institutional conditions, social inequalities and policy gaps. They also show that these costs are not inevitable consequences of aging alone; they are shaped by how societies organize support across families, communities, markets and public systems. As populations continue to age, future research and policy should move toward equitable, sustainable and person-centered models of care that recognize the diverse experiences of older adults and the shared responsibility required to support healthy aging.

